# Orbital fibroblastic reticular cell tumor: A case report and literature review for a rare disease

**DOI:** 10.1097/MD.0000000000042807

**Published:** 2025-06-06

**Authors:** Yu Yan, Shuangle Li, Mi Zhou, Qin Zhong

**Affiliations:** a Department of Ophthalmology, Zigong First People’s Hospital, Zigong, Sichuan Province, China; b Department of Ophthalmology, Mianyang Traditional Chinese Medicine Hospital, Mianyang, Sichuan Province, China.

**Keywords:** fibroblastic reticular cell tumor, imaging, immunohistochemistry, orbital tumor, pathology

## Abstract

**Rationale:**

This study investigates the clinical, imaging, and pathological features of fibroblastic reticular cell tumors (FRCTs) through a retrospective analysis of a patient with FRCT, along with a review of relevant literature.

**Patient concerns:**

A 49-year-old male was admitted to our hospital because of swelling and discomfort in the right eye, occasionally accompanied by double vision, for more than 3 months. Physical examination revealed an obliquely downward right eye, ptosis, and a palpable medium–hard tumor at the supraorbital rim of the orbit.

**Diagnoses:**

An orbital B-type ultrasound, orbital computed tomography, and orbital contrast-enhanced magnetic resonance imaging were performed, and the findings suggested a diagnosis of right orbital hemangioma.

**Interventions:**

Following imaging studies, the tumor was surgically excised. Microscopic pathological examination revealed that the lesion was composed of lymphatic follicles and spindle cells. Immunohistochemistry revealed that: the tumor is mainly composed of spindle fibroblastic cells, accompanied by the formation of lymphoid follicles. Immunohistochemical staining shows that the lymphoid follicles express CD20 positively, while the Ki67 positive index of the spindle tumor cells is lower. Based on these findings, the pathologists believed that the lesion was consistent with an FRCT.

**Outcomes:**

The patient refused subsequent treatment and was discharged. Postoperative imaging (computed tomography and magnetic resonance imaging) conducted at 4 and 24 weeks revealed no recurrence of the tumor.

**Lessons:**

FRCTs are exceedingly rare in clinical practice, This is the first case report of an orbital FRCT. The main clinical manifestation is a painless orbital mass, and the imaging findings are nonspecific; therefore, the diagnosis mainly depends on the pathology and immune phenotype of the tumor. Currently, there are no detailed data regarding the effects of postoperative adjuvant therapy. With more reports and studies on patients with FRCT, the diagnostic accuracy for this disease can be increased, and more accurate and personalized treatment plans can be developed.

## 1. Introduction

Fibroblastic reticular cell tumors (FRCTs) are malignant non-lymphocyte (reticular cell) growths^[1]^ that were first reported by Turner in 1984. FRCTs mainly develop in the lymph nodes of young people and adults, but may also occur in soft tissues, the mediastinum, and the spleen.^[2]^ Fibroblastic reticular cells (FRCs) are a subgroup of dendritic cells (DCs), a type of antigen-presenting cells (or immune helper cells). According to their morphology and immune phenotype, DCs can be divided into 4 types according to their morphology and immune phenotype: Langerhans cells, interdigitating DCs (IDCs), follicular DCs (FDCs), and FRCs.^[3,4]^ Tumors originating from DCs are rare, and those tumors originating from FRCs are even rarer in clinical practice. To the best of our knowledge, this is the first case report of an orbital FRCT. The clinical, radiological, and pathological features of a patient with an FRCT at our hospital were analyzed, and a literature review was conducted.

## 2. Case description

### 2.1. Clinical data

On May 20, 2023, a 49-year-old middle-aged male was admitted to our hospital for treatment of swelling and discomfort in the right eye, occasionally accompanied by double vision, for 3+ months. The patient had a 5-year history of hypertension, and his blood pressure was well-controlled with medication. The patient had a history of smoking and alcohol consumption. Specialist examination revealed a visual acuity of OD 1.0 and OS 1.0; the intraocular pressure was 16.0 mm Hg in the right eye and 12.0 mm Hg in the left eye. A color vision test showed that both eyes could distinguish between red and green. The right eye was inclined downwards and had limited upward rotation; eye movement in other directions was normal. Mild swelling and ptosis of the right eyelid were also observed. The palpebral margin was 2 mm below the corneal limbus. A medium–hard mass was palpable at the lateral margin of the orbit with poor mobility and a clear boundary. The mass measured approximately 1.0 × 1.0 × 0.5 cm, and extended into the orbit. Other observations of the right eye were as follows: head-dropping test (+), Valsalva maneuver (+), no congestion or edema of the conjunctiva, no jaundice or nodules in the sclera, clear cornea, normal corneal sensory test, negative keratic precipitates, anterior chamber depth of 3.0 corneal thickness, peripheral chamber depth of 0.5 corneal thickness, AR(‐), iris texture clear, normal color, round pupil with a diameter of 3 mm, sensitivity to light reflection, mild lens opacity, no vitreous opacity, clear optic disc boundary on the right fundus, C/D = 0.3, flattening of the retina, and presence of the macular reflex. No abnormalities were observed in the left eye.

### 2.2. Auxiliary examination

#### 2.2.1. Before admission

Orbital MRI (Ningbo Beilun District Hospital of Traditional Chinese Medicine) revealed a mass in the right lacrimal gland area, and further examination was recommended.

#### 2.2.2. After admission

##### 2.2.2.1. Random blood glucose: 10.2 mmol/L

**Figure 1.**
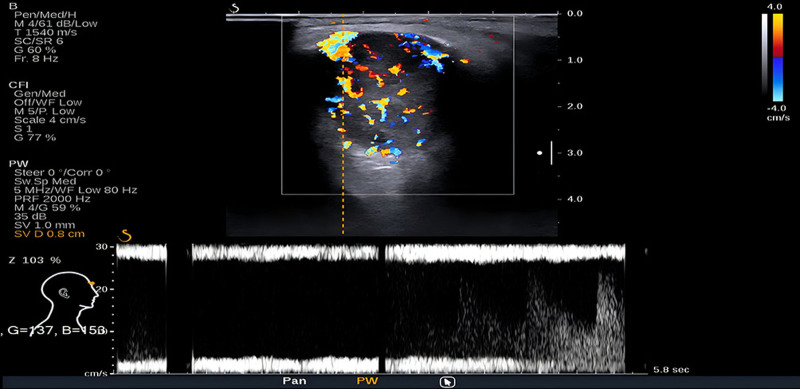
B-type ultrasound showed the size of mass is about 2.6 × 2.6 × 1.5 cm, the CDFI showed abundant blood flow signals. CDFI = color Doppler flow imaging.

B-type ultrasound of the superficial organs for the “mass” in the right orbit (Fig. 1) revealed a hypoechoic mass measuring approximately 2.6 × 2.6 × 1.5 cm, the lower end closely attached to the right eyeball. Color Doppler flow imaging showed abundant blood flow signals in and around the mass.

**Figure 2.**
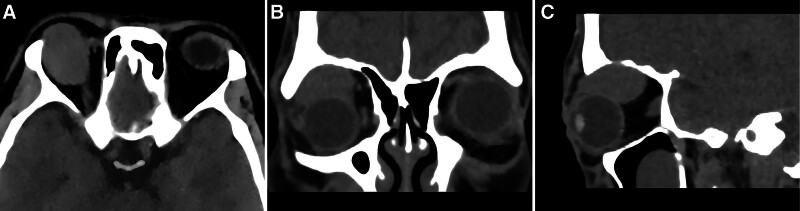
CT images. Transverse (A), Coronal (B), Sagittal (C). Suggested an oval soft tissue mass, unclear boundary with the anterior part of the superior rectus muscle, approximately 3.1 × 2.3 × 2.4 cm in size. CT = computed tomography.

Computed tomography (CT) of the orbit (Fig. 2) revealed an oval soft tissue shadow above the right eyeball, an unclear boundary with the anterior part of the superior rectus muscle, a maximum diameter of approximately 3.1 cm; excessive pressure on the right eyeball, normal bilateral eyeball shapes, no abnormal density shadow inside the eyeball, and a clear intra-orbital fat gap. The diagnostic results indicated that the nature of the soft tissue shadow above the right eyeball was determined. The possibility of a benign lesion possibly originating from the superior rectus muscle was considered, although other sources were not excluded. Therefore, further examination is recommended.

**Figure 3.**
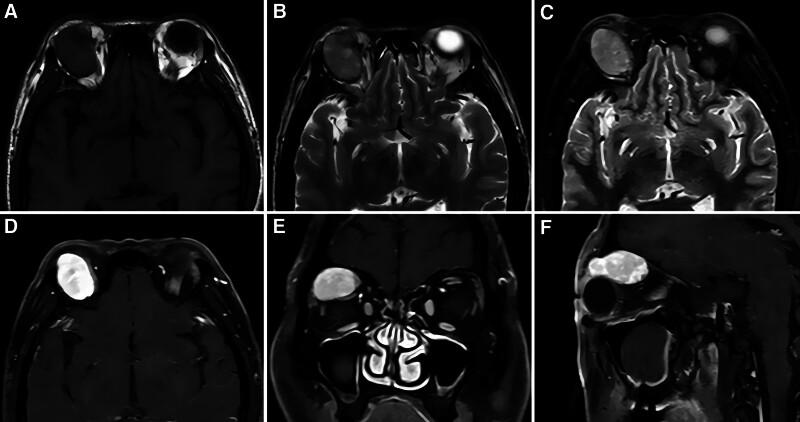
CE-MRI images. T1WI, the tumor was hypointense (A); T2WI, the tumor was slightly hyperintense (B); T2WI–SPAIR showed high signal (C); CE-MRI showed significant uneven enhancement of the tumor (D–F). CE-MRI = contrast-enhanced magnetic resonance imaging.

Orbital contrast-enhanced magnetic resonance imaging (CE-MRI) (Fig. 3) revealed an oval, soft tissue mass next to the superior rectus muscle in the extraconal orbital space of the right orbital muscle, approximately 2.4 × 3.1 × 1.9 cm in size, with a clear boundary and a uniform signal (isointense on T1 weighted image [T1WI] and slightly hyperintense on T2 weighted image [T2WI] and STIR imaging). The contrast-enhanced scan revealed fine, uniform, persistent enhancement, and compression of the right superior rectus muscle. The extraocular muscles and lacrimal glands were not enlarged, and the courses of the optic nerve and optic chiasm were normal. The diagnosis was a mass next to the superior rectus muscle in the extraconal space of the right orbital muscle. The patient was diagnosed with a benign tumor (cavernous hemangioma).

### 2.3. Treatment

There were no characteristic results from the preoperative examinations. The disease condition was explained to the patient, and pathological examination after surgical resection was recommended to confirm the diagnosis and formulate a treatment plan. After induction of general anesthesia, a surgical incision was made through the conjunctival approach. The conjunctiva of the superior fornix was incised to approximately 2 cm in length, and the subconjunctival tissue was bluntly dissected. The orbital septum was cut open, adipose tissue around the tumor was isolated and exposed, and the orbital fat was compressed with a spatula. The tumor was bluntly separated from the surrounding tissue adhesions via endoscopy assistance, completely exposed, and then removed. The conjunctiva was reduced and closed using 10-0 sutures. The eye was then smeared with ofloxacin ointment, cleaned, and bandaged. Intraoperative findings revealed that the tumor was located between the superior rectus and levator palpebral muscles. The tissue was solid, medium–hard, and fragile, with a gray–white–red color, a size of approximately 2.6 × 2.0 × 1.5 cm in size, a smooth surface, and a partial capsule. The incisal surface was gray–white, gray–red, and honeycomb-shaped, similar to a Manson tumor. The samples were sent for pathological examination.

### 2.4. Pathological examination results

**Figure 4.**
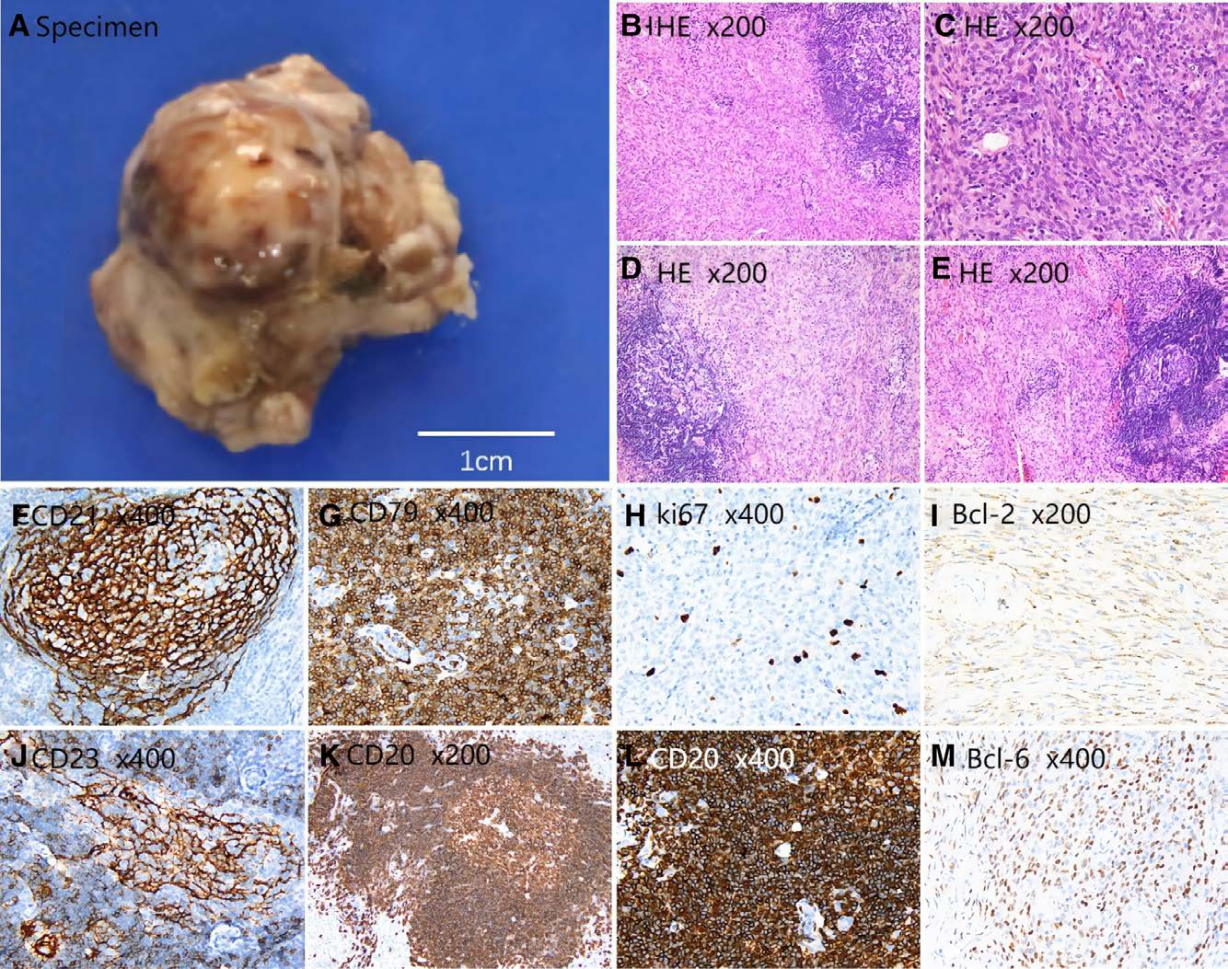
Specimen (A). Histology: hematoxylin and eosin (HE) (B–D) and (F–M) some of the most relevant immunohistochemical stains (F–M). Magnification ×200 or ×400.

The right orbital tumor was a gray–white and gray–red piece of nonplastic tissue, solid, medium–hard, fragile, 3 × 2.3 × 1.7 cm in size, with a smooth surface and a partial capsule. The incisal surface was gray–white and gray–red. It was difficult to determine the nature and histological type of the lesion through pathological examination in our hospital, so it was recommended that the patient undergo telepathology for further diagnosis.Opinion of telepathology from West China Medical Center, Sichuan University: Microscopic examination of the <right orbital tumor> (Fig. 4) indicated that the lesion was composed of lymphoid follicles and spindle cells. Small lymphocytes were scattered among the spindle cells. Immunohistochemistry (IHC) revealed that the lymphatic follicles were cluster of differentiation (CD) 20+ and CD79a+, and the germinal centers were CD10+, B-cell lymphoma (BCL) 2−, BCL6+, and c-Myc−. The percentage of Ki67-positive cells was ~70%. The FDC networks were CD21+, CD23+, CD35+, and D2‐40+. The spindle cells were CD21‐, CD23‐, CD35‐, S100‐, BCL2+, BCL6+, cyclic Cl‐, desmin+ (weak), SMA‐, Caldesmon‐, PCK+ (partially weak), and D2‐40‐. The percentage of Ki67-positive cells was approximately 5%. Background: Small lymphocytes were CD3+, CD5+, and CD1a‐. CD68+ cells were scattered throughout the tissue samples. The tumor is mainly composed of spindle fibroblastic cells, accompanied by the formation of lymphoid follicles. Immunohistochemical staining shows that the lymphoid follicles express CD20 positively, while the Ki67 positive index of the spindle tumor cells is lower.Based on these findings, the pathologists considered the lesion to be consistent with an FRCT.

### 2.5. Results and follow-up

The patient recovered well after the surgery. After consultation with the oncology department, the patient refused further treatment and was discharged. The patient was very satisfied with the results of the treatment and presented a banner as a token of appreciation. CT and MRI examinations of the orbit at 4 and 24 weeks after surgery revealed no recurrence.

Our study was exempted from review, and this exemption complied with the policies of our institutional review board. Written informed consent was obtained from the patient for publication of this report and accompanying images.

All relevant data are within the paper and its supporting information files.

## 3. Discussion

### 3.1. Fibroblastic reticular cells

Antigen-presenting cells (or immune helper cells) present antigens to T and B cells and include various types of cells, such as DCs and macrophages. DCs are a subset of non-lymphocyte cells also known as reticulocytes. DCs can be divided into 4 types according to their morphology and immune phenotype: Langerhans cells, IDCs, FDCs, and FRCs.^[5]^ FRCs are usually located in the capsule, porta, and mesenchymal areas of lymph nodes, that is, the T-cell area,^[6]^ and are wrapped around capillaries in the lymph nodes, but some are found in other locations, including the parafollicular regions of the spleen and tonsils. FRCs form an FRC network that is responsible for transferring lymph fluid from the tissue to the T-cell area through afferent lymphatic vessels, promoting the secretion of various cytokines and other regulatory factors, affecting the immune response, and controlling the proliferation of lymphocytes.^[6,7]^

### 3.2. Fibroblastic reticular cell tumors

According to the World Health Organization classification, DC tumors are rare and commonly include FDC sarcomas (FDCSs), IDC sarcoma, and FRCTs.^[8]^ Based on cytokeratin expression, FRCTs can be further subdivided into cytokeratin-negative and cytokeratin-positive interstitial reticulum cell (CNRC and CIRC, respectively) tumors.^[9]^ Accurate diagnosis of FRCTs is difficult, particularly when they are located in extralymphatic locations. Furthermore, these tumors must be differentiated from other common tumors, including carcinomas and soft tissue sarcomas.

### 3.3. Clinical features

FRCT is most common in the lymph nodes but can also occur in the mediastinum, soft tissues, kidneys, adrenal glands, spleen, lungs, and breasts.^[10]^ The manifestations of FRCTs are more similar to those of low-grade sarcomas than malignant lymphoma.^[11]^ The initial manifestation is a painless tumor that can be palpated on the body surface.^[12]^ In this case, the main symptoms of the patient were ocular swelling and discomfort, a medium–hard, painless mass palpable at the supraorbital rim, and a positive head-dropping test and Valsalva maneuver, similar to the first manifestations of a painless tumor. In such cases, imaging examinations should be subsequently performed to rule out orbital hemangiomas, lymphomas, and lacrimal gland tumors.

### 3.4. Imaging features

The imaging manifestations of FRCTs are nonspecific.^[11]^ An analysis of the literature revealed multiple reports of FRCTs of the thorax, mediastinum, and internal iliac lymph nodes. A combination of noncontact fluorescence and X-ray computed tomography revealed tightly arranged tumor cells, a rich blood supply, and a tumor appearance similar to a pseudoaneurysm. Contrast-enhanced CT shows obvious enhancement, and the internal patchy and stripe-like low-density shadows are considered collagen fiber components in the tumor. Hyperintense signals on T1WI and hypointense signals on T2WI can be characteristic manifestations of FRCTs in spleen; on color Doppler examination, inflammatory pseudotumor-like FDCSs appear as hypoechoic lesions, with enhancement similar to that observed on CT.^[13]^ Inflammatory pseudotumor-like FDCS have abundant small blood vessels and capillaries.^[12,14]^ The color Doppler examination results of the present case are in agreement with those described in the literature. On both CT and CE-MRI, the orbital tumor in the patient presented as a uniformly enhanced signal. CE-MRI showed a clear lesion, uniform boundary, isointense signal on TIWI, slightly hyperintense signal on T2WI and STIR imaging, and uniform, continuous enhancement on enhanced imaging. We speculated that the uniform and high-density CT manifestations were due to the rapid growth of FRCTs, density of the tumor cells, and their rich blood supply. The manifestations on plain and CE-MRI were similar to those of the cavernous vessels. Before surgery, the radiology department of our hospital suspected orbital cavernous hemangioma. Our analyses showed that the tumor originated from FRCs, which are mostly present in the paracortical area of the lymph nodes and surround the high endothelial venules, forming a complex reticular structure.^[15]^ Subsequent imaging studies revealed characteristics of rich blood flow, which can be easily confused with those of cavernous hemangiomas.

CT can clearly show the tumor location, density, size, morphology, and relationship with adjacent tissues and can therefore be used to accurately diagnose most orbital tumors, especially benign tumors.^[16,17]^ Hou et al^[18]^ achieved a diagnostic accuracy of 0.9113, sensitivity of 0.8684, specificity of 0.9302, and area under the curve of 0.9535 for diagnosing orbital cavernous hemangioma with T1WI. Magnetic resonance diffusion-weighted imaging (MR-DWI) is a noninvasive method for observing the microscopic, diffuse movement of water molecules in human tissues. It can be used to detect lesions before any morphological changes occur and that are not detectable with conventional MRI scans. Diffusion-weighted imaging (DWI) does not require contrast agent administration, is simple to perform, and has a good reproducibility. Lesions can be quantitatively analyzed by measuring their apparent diffusion coefficient (ADC). A more accurate preoperative DWI sequence and determination of the ADC can guide clinical practice, help differentiate benign from malignant lesions, and play an important role in the diagnosis and treatment selection for orbital tumors.^[19,20]^ Unfortunately, owing to a lack of experience, the preoperative examination was completed in this case without further MR-DWI and ACD measurements.

Currently, multimodal quantitative CE-MRI and DWI are used for the identification and staging of breast cancer and cervical cancers.^[21,22]^ Special sequences, such as MR-DWI, can be used for FRCT because different tumors exhibit different restricted diffusion characteristics on DWI, as well as different ADC values. However, more cases and corresponding imaging data are needed to leverage the differences in the DWI findings and ADCs of the different tumors to establish models and obtain auxiliary evidence for the differential diagnosis of FRCTs. Moreover, a combination of CT and MRI can provide a more accurate diagnosis before surgery, aid in formulating a better surgical plan, and guide follow-up treatment after surgery.

### 3.5. Histopathological features

After complete tumor removal, the morphology was found to be similar to that of Masson’s tumors (intravascular papillary endothelial hyperplasia), and the diagnosis was confirmed by pathological and immunohistochemical examinations. IHC-specific indicators of FRCT include desmin(+), SMA(+), CD21(‐), CD1a(‐), CD35(‐), and S100(‐).^[9,23,24]^ Studies have shown that lymph node B-type ultrasound, CT, bone marrow morphology, and biopsy examinations are insufficient for diagnosing FRCT, but have important differential diagnostic value.^[25]^ Histopathologically, FRCT should be differentiated from the following tumors:^[26,27]^ (1) FDC tumors; (2) IDC tumors; (3) intranodal palisaded myofibroblastomas; (4) lymphomas; (5) sarcomas; and (6) inflammatory myofibroblastic tumors.

Treatment: FRCTs are rare, and there are no detailed data on the efficacy of postoperative adjuvant therapy.^[14]^ Yin et al^[28]^ collected data from 23 FRCT patients in China and abroad whose lesions were located in different locations. Among the 20 patients who returned for follow-up, almost all underwent surgery, some received adjuvant radiotherapy and chemotherapy, and 6 patients developed tumor recurrence and metastasis within 2 years, with a primary tumor recurrence rate of 9.1%, a metastasis rate of 36.4%, and an overall mortality rate was higher than 25%. After explaining these results to the patient, he chose to temporarily stop subsequent treatments, including chemotherapy and radiotherapy, and is currently being closely monitored in an outpatient clinic.

## 4. Conclusion

FRCTs are rare. This is the first case report of an FRCT originating from an orbit. This tumor most commonly develops in the lymph nodes but may occur in the soft tissues, mediastinum, or spleen, and only 1 case report has described the onset of FRCT in the subcutaneous tissue of the eyelid.^[29]^ In the present case, the manifestations included a painless orbital mass and a positive head-dropping test and Valsalva maneuver. Preoperative imaging findings and tissue characteristics cannot provide a clear diagnosis; therefore, postoperative pathological biopsy and IHC were performed to confirm the diagnosis. The incorporation of multimodal, quantitative MRI and DWI would allow a comprehensive analysis of the clinical manifestations and imaging data that, combined with pathology (gross and IHC examinations), could improve the preoperative diagnosis and provide a more complete basis for surgical planning and postoperative follow-up and treatment. In this case, DWI was not performed before surgery; therefore, no more data were available. For similar cases in the future, clinicians, radiologists, and pathologists need to work closely to comprehensively evaluate the imaging features, patient’s medical history, physical examination findings, other ancillary test data, and pathologic IHC data to jointly formulate an appropriate diagnosis and treatment plan. Although studies on the clinical features, diagnosis, treatment, and prognosis of FRCT have resulted in a preliminary understanding of the disease, many questions remain and further research is needed. Currently, surgical resection is the main treatment, but it is not clear whether postoperative adjuvant radiotherapy and chemotherapy can be used to reduce the recurrence rate. As more FRCT cases are reported, more clinical, imaging, and pathological data will be available for further analysis. Taking advantage of the rapid development of imaging technology, the integrated application of multimodal imaging would provide new prospects for the differential diagnosis of this rare disease and offer the potential to increase diagnostic accuracy and reliability, thus allowing the development and provision of more accurate and individualized treatment plans.

## Acknowledgments

We would like to thank the telepathology data from West China Medical Center and the selfless sharing of the patients.

## Author contributions

**Conceptualization:** Yu Yan, Mi Zhou.

**Data curation:** Yu Yan, Qin Zhong.

**Investigation:** Yu Yan, Qin Zhong.

**Methodology:** Yu Yan, Shuangle Li.

**Resources:** Yu Yan.

**Supervision:** Shuangle Li.

**Visualization:** Yu Yan, Shuangle Li, Mi Zhou, Qin Zhong.

**Writing – original draft:** Yu Yan.

**Writing – review & editing:** Yu Yan, Shuangle Li.
